# Editorial: Women in Neuroanatomy

**DOI:** 10.3389/fnana.2023.1343539

**Published:** 2023-12-12

**Authors:** Lidia Alonso-Nanclares

**Affiliations:** ^1^Instituto Cajal, Consejo Superior de Investigaciones Científicas, Madrid, Spain; ^2^Laboratorio Cajal de Circuitos Corticales, Centro de Tecnología Biomédica, Universidad Politécnica de Madrid, Pozuelo de Alarcón, Madrid, Spain

**Keywords:** neuroscience, women, gender equity, brain, neuroanatomy

In the field of Neuroscience, according to the US Society for Neuroscience, the proportion of women and men is relatively equal. However, there is a lack of representation of women in senior positions—comprising < 14% of the tenured neuroscience faculty. Despite the embarrassing fact that women represent a minority of the neuroscience faculty, many women are contributing to the field and tackling important questions.

Dworkin et al. (2020) found that neuroscience reference lists tend to include more papers with men as first and last authors than would be expected if gender were not a factor. Data from the Women in Neuroscience Repository show that women: (i) publish significantly fewer papers as first or last contributors than men; (ii) are awarded significantly fewer prizes; and (iii) appear significantly less as speakers in departmental seminar series and conferences (Schrouff et al., 2019). Recently, Ross et al. (2022) found that women in research teams are significantly less likely than men to be credited with authorship in most scientific fields and at almost all career stages.

As a specific action for supporting women neuroscientists, the present Research Topic includes 9 articles in which the first or last authors are women in the specialized field of Neuroanatomy. The formats include original research, review and perspective articles, and range across a variety of methods and species. The small but representative number of articles included in this volume are briefly summarized below:

Zhang et al. present a primary explorational study using T3 MRI analysis on individuals residing on the high-altitude Tibetan plateau. Their data suggest that while the overall hippocampal volume is not affected, the core hippocampus of Tibetans may be adapted to chronic hypobaric hypoxia. However, this adaptation may have required generations—rather than decades—to accumulate in the population.

Navarro-López et al. investigates the specific temporal pattern of hippocampal cell adhesion molecule expression during the memory acquisition process, combining conditioning training, electromyography recording, and ELISAs. Their findings highlight the relevance of specific neural cell adhesion molecules (L1, PSA-NCAM, and NCAM) as learning-modulated molecules critically involved in remodeling processes underlying associative motor-memory formation.

Using immunohistochemistry techniques, Cisneros-Larios and Elias report that the posterior nucleus of the amygdala is the main site where specific neurons (those expressing *prokineticin receptor 2*) may regulate aspects of the reproductive function and social behavior in adult mice.

Ábrahám et al. study the postnatal developing human hippocampal formation using immunohistochemistry. They report that maturation of PV-immunoreactive interneurons follows the developmental sequence of the subfields of the human hippocampal formation, providing morphological evidence for the long-lasting functional maturation of the human cortex.

Baizer and Witelson compare neuroanatomical and neurochemical organization of four human brainstem nuclei to nuclei in other mammals including chimpanzees, monkeys, cats and rodents. Their results suggest several principles of human brainstem organization that distinguish humans from other species.

Masse et al. used fetal *in vivo* structural T2-weighted MRI to characterize the differences in spatiotemporal brain maturation between Chiari II malformation and normal human fetal controls. They found significantly smaller volumes of the diencephalon and significantly larger volumes of lateral ventricles and proliferative zones in the fetuses with this malformation. They concluded that regional brain development should be taken into consideration when evaluating prenatal brain development in fetuses affected by Chiari II.

Martínez-Gil et al. review the molecular and morphological changes in retinal cells that occur in response to oxidative stress and the inflammatory processes underlying inherited retinal dystrophies (a group of genetic disorders with a prevalence of 1 in 3,000 individuals with no efficacious treatment to date). They suggest that a deep knowledge of the molecular mechanisms involved in retinal degeneration will hopefully reveal suitable targets for the development of therapeutic molecules.

The review by Brewer and Barton discusses the evidence for cortical field maps and cloverleaf cluster organization across the human sensory cortex, as well as approaches used to identify such organizational patterns. Knowledge of how these topographical representations are organized across the cortex provides us with insight into how our conscious perceptions are created from our basic sensory inputs. Studying these representations serves as an important tool for developing improvements in clinical therapies and rehabilitation for sensory deficits.

The perspective article by Salcedo-Arellano et al. reflects on the success stories of four Hispanic women neuroscientists who have opened doors for many of us. The paper highlights their great contributions by recognizing the outstanding work they have done, and continue to do, in identifying anatomical, molecular and cellular mechanisms that underlie normal and pathological processes in the brain.

In summary, this volume brings together a series of articles dealing with the development of new methods and approaches to be applied in modern neuroanatomy, as well as reflections on recent findings and a historical perspective of women's achievements in neuroanatomy. The Research Topic has attempted to recognize the breadth of scientific ideas and findings. Further, even within this small sample, it aims to spotlight women's contribution to the scientific effort at large.

## Author contributions

LA-N: Writing—original draft, Writing—review & editing.
